# Effects of Pavement Surface Texture and Interfacial Friction on Tire Rolling Resistance: A Finite Element Study

**DOI:** 10.3390/ma19204418

**Published:** 2026-10-20

**Authors:** Quanjun Shen, Xiaodong Zhou, Fusen Zheng, Tianxiang Xie, Shaojie Gao, Dongliang Hu

**Affiliations:** 1 Shandong Hi-Speed Group Innovatio6n Research Institute, Jinan 250014, China; 2 Dongguan Municipal Urban Construction Management Bureau, Dongguan 523000, China; 3 School of Highway, Chang’an University, Xi’an 710000, China; 4 Beijing Foton Daimler Automotive Co., Ltd., Beijing 101400, China

**Keywords:** tire–pavement interaction, rolling resistance, pavement surface texture, friction coefficient, energy dissipation, finite element analysis

## Abstract

To investigate the effects of pavement textures and tire–pavement interfacial friction on tire rolling resistance, a three-dimensional tire–pavement rolling finite element model was developed considering flat, arc-shaped, triangular, and rectangular textures under different friction coefficient conditions (*μ* = 0.2, 0.4, 0.6, and 0.8). The effects of textures and *μ* on tire–pavement contact responses, energy dissipation, and rolling resistance were analyzed, and the rolling resistance coefficient (*C_rr_*) was calculated based on energy dissipation theory. Furthermore, scaled rolling resistance tests were conducted under different pavement surface materials to validate the trends obtained from finite element simulations. The results show that pavement textures significantly affect tire–pavement contact responses under rigid pavement conditions. Compared with the flat texture, the triangular texture increases the contact length and peak contact pressure by approximately 18.9% and 458.1%, respectively, indicating enhanced contact pressure concentration. Compared with rigid pavement, flexible pavement structural deformation further influences tire–pavement contact responses and extends the deformation region. The arc-shaped texture exhibits more pronounced deformation responses and higher energy dissipation. As *μ* increases from 0.2 to 0.8, both tire deformation energy dissipation and tire–pavement interfacial frictional dissipation increase, resulting in higher *C_rr_*. *C_rr_* increases by approximately 14.5–30.8% and 10.4–16.2% for rigid and flexible pavements, respectively. Scaled test results show that the trends in rolling resistance coefficients for different pavement materials are consistent with surface friction levels, and additional loading further increases rolling resistance. The results demonstrate that pavement textures and tire–pavement interfacial friction both influence rolling resistance through their effects on contact behavior and energy dissipation, providing a theoretical basis for low-rolling-resistance pavement design.

## 1. Introduction

With increasing global energy consumption and growing environmental concerns, improving the energy efficiency of transportation systems has become an effective strategy for reducing energy consumption and emissions [1]. Tire rolling resistance represents a major source of energy loss during vehicle operation, directly affecting fuel economy and the driving range of electric vehicles [2,3]. Thus, elucidating the mechanisms governing rolling resistance is essential for the development of energy-efficient vehicles and pavement design [4]. From an energy dissipation perspective, rolling resistance is associated with energy losses within the tire–pavement system, primarily due to viscoelastic hysteresis loss in rubber materials [5] and tire–pavement interfacial frictional dissipation [6]. Viscoelastic hysteresis arises from the phase lag between stress and strain during cyclic deformation of rubber, resulting in irreversible energy dissipation, which is largely converted into heat [7].

Previous studies on rolling resistance have primarily focused on the effects of rubber viscoelastic properties, tire structural design, and operating parameters [8]. In recent years, advances in computational mechanics have enabled finite element simulations of tire rolling behavior, which provide an effective approach for investigating tire deformation responses and energy dissipation mechanisms [9,10]. Finite element simulations based on nonlinear tire models considering rubber constitutive behavior, reinforcement structures, and dynamic tire–pavement rolling contact behavior can effectively capture tire stress distributions, deformation responses, and energy dissipation characteristics under different tire–pavement contact conditions, thereby elucidating the energy dissipation mechanisms during tire–pavement contact [4,11,12,13]. At present, rolling resistance measurements are mainly conducted through full-scale vehicle tests and laboratory bench tests. Full-scale vehicle tests can reflect actual operating conditions but are susceptible to changes in the surrounding environment and pavement conditions, while also involving relatively high testing costs [14,15]. Laboratory tests offer better controllability and repeatability; however, their controlled test conditions make it difficult to reproduce complex tire–pavement contact behavior under realistic pavement conditions [16]. Overall, the finite element method provides an effective approach for analyzing rolling resistance mechanisms under complex tire–pavement interactions.

However, existing studies on rolling resistance have primarily focused on tire-related factors, whereas the effects of pavement surface textures and friction characteristics remain insufficiently understood [8,17]. Real pavement surfaces exhibit complex textures, whose geometric configurations, texture scales, and interfacial friction characteristics can significantly influence tire–pavement contact behavior, pressure distributions, deformation responses, and energy dissipation processes [18,19]. Despite the importance of pavement textures, related investigations have mainly focused on macroscopic performance indicators such as skid resistance [20], while the effects of pavement texture geometries on tire–pavement contact behavior and the underlying mechanisms governing rolling resistance remain insufficiently understood [21]. Previous studies have incorporated pavement texture characteristics into three-dimensional tire–pavement interaction models. Contact behavior on patterned tire–asphalt pavement has been investigated [9,22], and the effects of pavement-related parameters, including texture characteristics, on tire–pavement contact and vibration responses have also been examined [23]. However, the combined effects of pavement surface geometry and interfacial friction on contact response, energy dissipation, and rolling resistance, particularly under both rigid and flexible pavement conditions, remain insufficiently understood. Therefore, a tire–pavement interaction framework that incorporates pavement surface texture, interfacial friction, and pavement deformability is needed to clarify their effects on rolling resistance.

**Figure 1:**
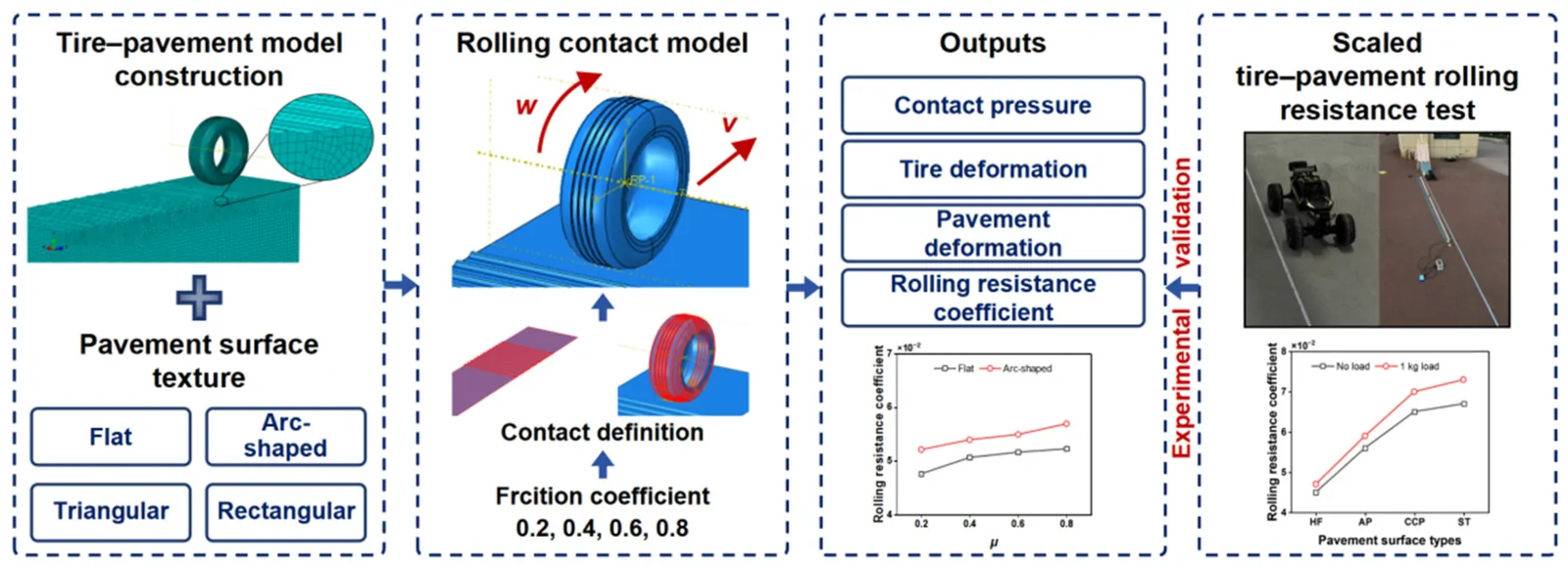
Research workflow.

Based on these considerations, this study developed a three-dimensional (3D) tire–pavement rolling finite element model with realistic tire structures, various pavement texture geometries, and rigid and flexible pavement conditions. Scaled rolling resistance tests were further conducted to validate the trends obtained from the finite element analysis. The research workflow is illustrated in Figure 1. The effects of texture geometries and interfacial friction on tire–pavement contact responses, energy dissipation, and rolling resistance were investigated to provide insights into the formation mechanisms of rolling resistance. The findings contribute to a better understanding of tire–pavement interactions and provide guidance for low-rolling-resistance pavement design.

## 2. Methodology

### 2.1. Tire–Pavement Finite Element Model

#### 2.1.1. Tire Model

**Figure 2:**
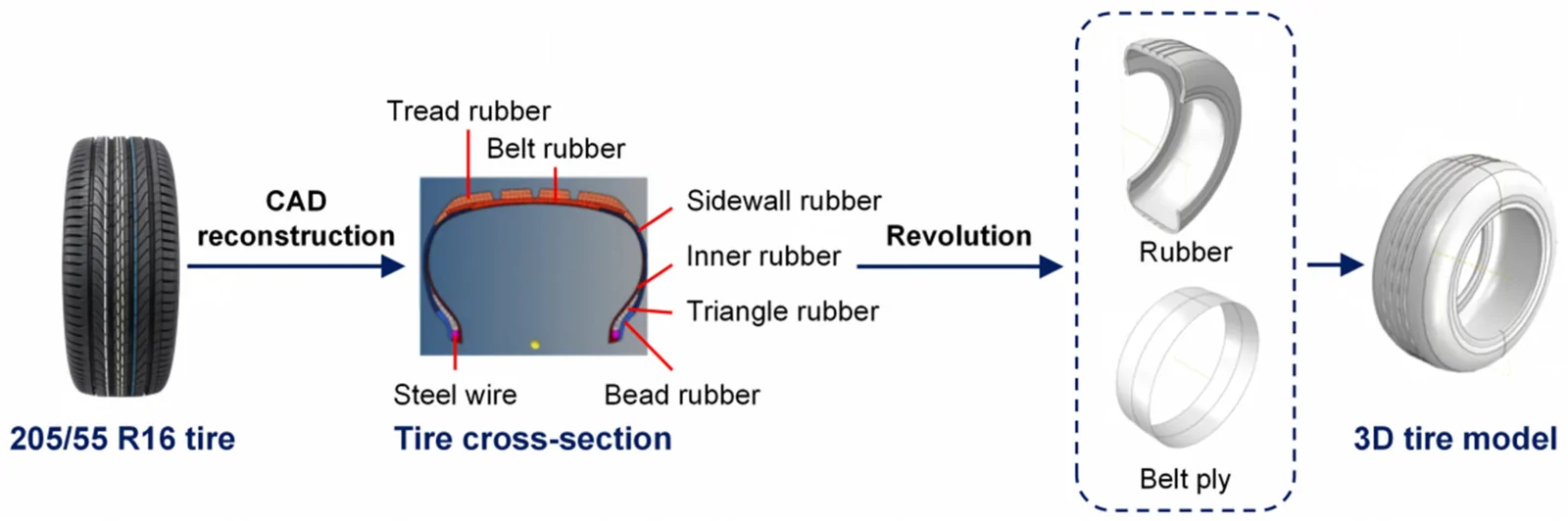
3D tire finite element modeling process.

To characterize tire–pavement contact behavior during rolling, a three-dimensional (3D) finite element model of an Ultra Contact 205/55 R16 radial tire was developed in ABAQUS. The 3D model was adopted to capture the non-uniform contact and deformation responses induced by pavement surface textures during tire rolling. Viscoelastic hysteretic loss was not introduced as a separate energy term in the present formulation. The selected tire is representative of light commercial vehicle applications and has broad engineering applicability. The two-dimensional (2D) cross-sectional geometry of the tire was experimentally measured and used to construct a reference 2D profile in AutoCAD 2024. The 2D profile was then imported into ABAQUS, and the 3D tire model was constructed by rotational sweeping, as shown in Figure 2. The rubber materials were modeled using the Yeoh constitutive model as Equations (1) and (2), with the corresponding material parameters given in Table 1. The tire reinforcement materials were represented using the Rebar model, with the relevant parameters listed in Table 2 [23]. For mesh discretization, the rubber materials were modeled using C3D8RH eight-node hybrid hexahedral elements, while the reinforcement materials were modeled using SFM3D4R four-node surface elements. To balance computational efficiency and mesh accuracy, mesh seed densities of 3 and 10 were adopted for the rubber and reinforcement materials, respectively, resulting in 430,272 and 32,220 elements. Identical mesh settings were adopted for all simulation cases to maintain consistent numerical resolution. Further assessment of the mesh sensitivity of peak contact pressure and *C_rr_* will be considered in future work.

(1) $$\begin{array}{c} U=C_{10}(I-3)+C_{20}(I-3{)}^{2}+C_{30}(I-3{)}^{3}+W \end{array}$$

(2) $$\begin{array}{c} I=\lambda_{1}^{2}+\lambda_{2}^{2}+\lambda_{3}^{2} \end{array}$$

where *U* is the strain energy density; I is the material twist metric; *C*_10_, *C*_20_, and *C*_30_ are the material parameters; *λ*_1_, *λ*_2_, and *λ*_3_ are the principal stretch ratios; and *W* is the volumetric strain energy, with *W* = 0 in this study.

#### 2.1.2. Pavement Model

**Figure 3:**
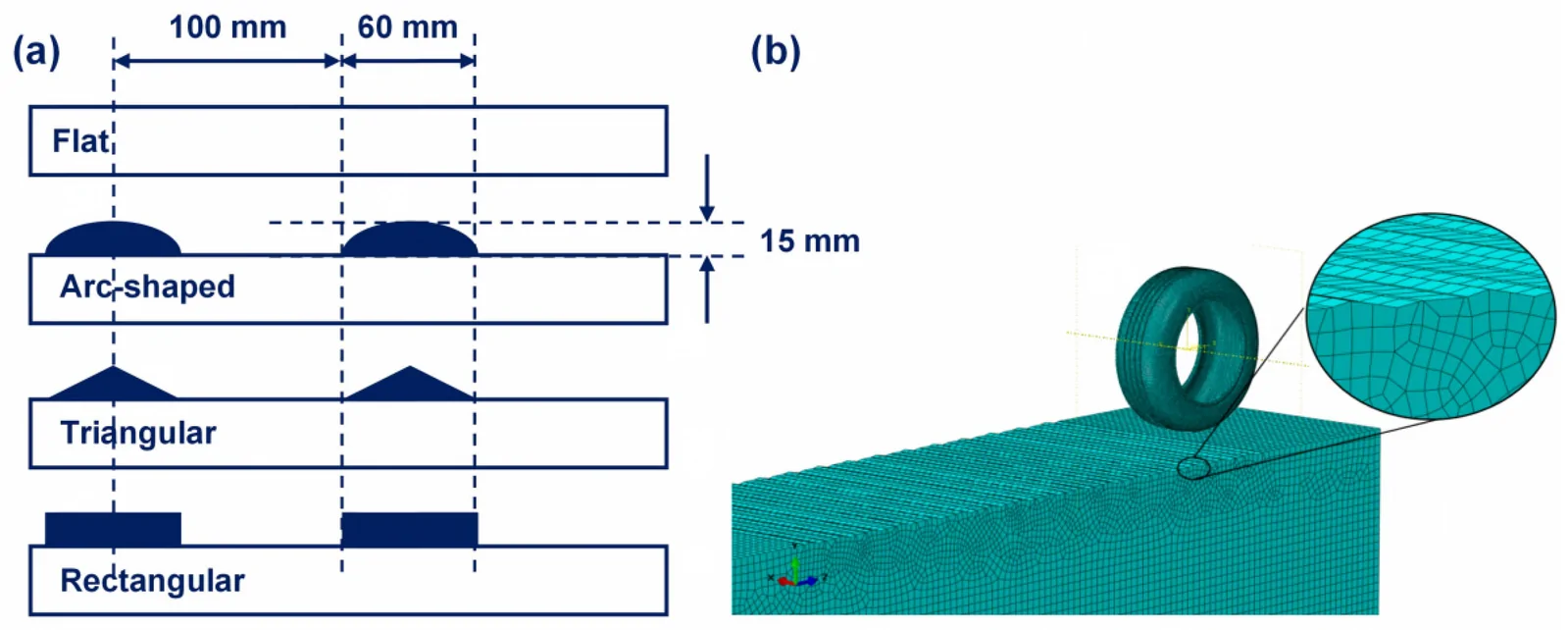
Pavement model: (**a**) surface textures; (**b**) 3D mesh of flexible pavement.

To evaluate the effects of pavement deformation on tire–pavement contact and rolling resistance, rigid and flexible pavement models were established. The rigid pavement was modeled as a 3D rigid body, while the flexible pavement was represented as a deformable three-layer structure consisting of a surface layer, a base layer, and a subgrade to capture deformation and stress transfer under tire loading. The material parameters of the pavement layers were determined based on JTG D50—2017 and previous studies and are listed in Table 3 [23]. In addition, four pavement surface geometries—flat, arc-shaped, triangular, and rectangular—were modeled (Figure 3). A texture height of 15 mm was adopted for the idealized surface geometries based on the macrotexture dimensions reported in the literature [24]. This value was selected to ensure consistent comparison among different surface geometries. The flexible pavement was discretized using solid elements, as shown in Figure 3b.

#### 2.1.3. Rolling Contact Model

**Figure 4:**
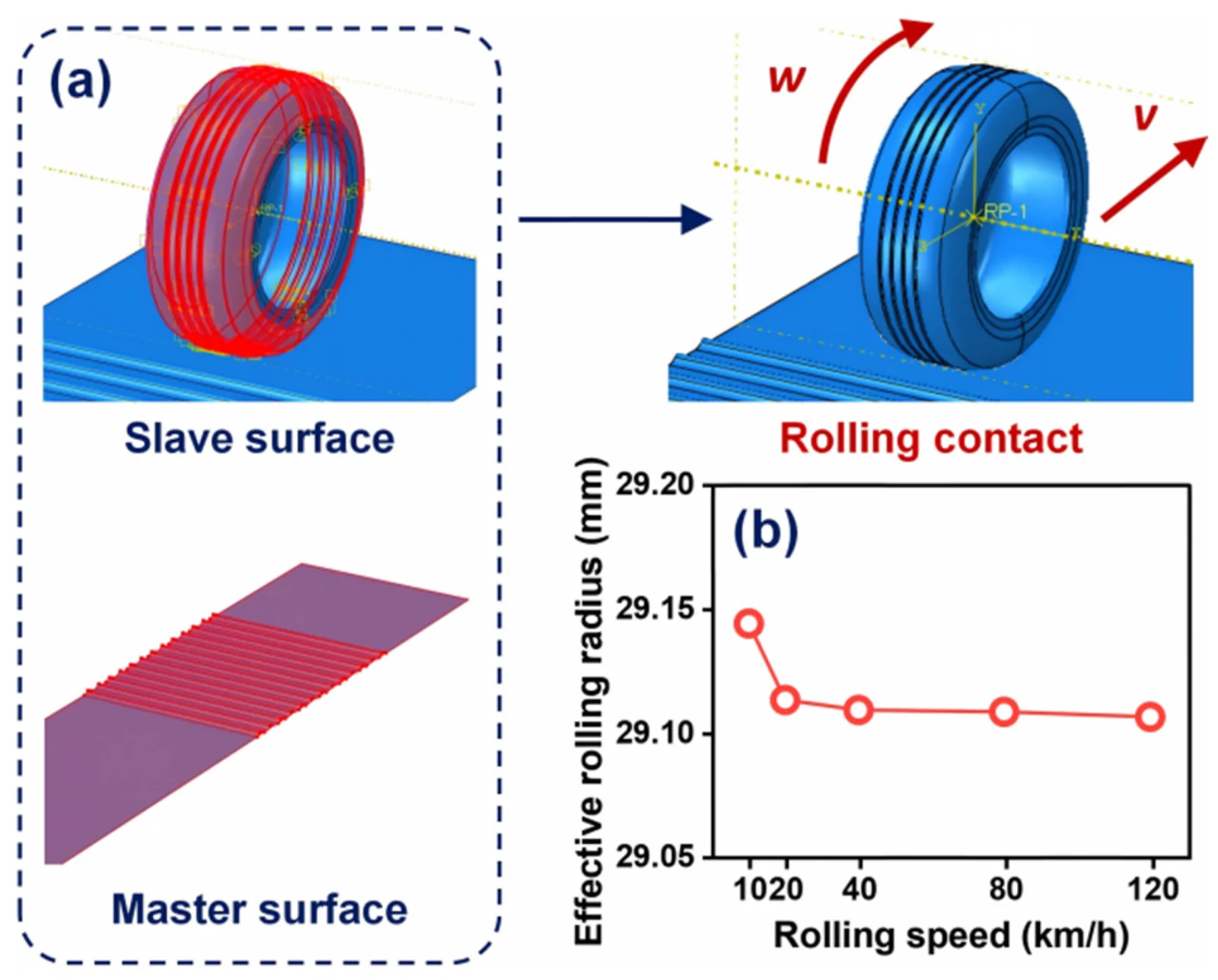
Tire–pavement rolling contact model: (**a**) contact definition; (**b**) *R_d_* with rolling speed.

The tire and pavement models were subsequently assembled within the same global coordinate system. Surface-to-surface contact with finite sliding was adopted at the tire–pavement interface to allow for continuous movement of the contact patch during rolling. According to the master–slave contact formulation, the pavement surface was assigned as the master surface and the outer tire surface as the slave surface, as shown in Figure 4a. For the normal contact behavior, the augmented Lagrange method was used to enforce the non-penetration constraint, while separation of the contact surfaces was allowed upon unloading. The allowable penetration was set to 0.01 mm. The tangential contact behavior was defined using the Coulomb friction model, with friction coefficients (*μ*) of 0.2, 0.4, 0.6, and 0.8 considered to evaluate the effects of interfacial friction on tire–pavement contact and rolling resistance.

To establish the tire–pavement rolling contact model, all degrees of freedom of the rim were initially constrained, and the tire inflation pressure was gradually increased from 0 to 0.25 MPa under quasi-static conditions until equilibrium was reached. The pavement was then moved toward the tire at 0.5 mm/s under displacement control to establish the initial tire–pavement contact. Subsequently, the displacement loading was terminated, and a vertical load was applied at 5 kN/s until a stable contact state was reached. Finally, translational (*v*) and angular (*ω*) velocities were applied at the wheel center along the travel direction and about the wheel axis, respectively, to simulate tire rolling.

To ensure consistency between v and ω during tire rolling, free-rolling simulations were first conducted on a flat rigid pavement to determine the effective rolling radius (*R_d_*) at different speeds. R_d_ was calculated using Equation (3) based on the mean wheel-center trajectory. Based on the free-rolling results, the absolute longitudinal creepage remained below 0.14% over the speed range of 10–120 km/h and was approximately 0.06% at 80 km/h, indicating that the tire remained close to a free-rolling state. *R_d_* reflects changes in the actual rolling distance caused by tire inflation, loading, and rolling deformation and was used as a key parameter in the subsequent calculation of rolling resistance. As shown in Figure 4b, *R_d_* decreased by only 0.13% over the speed range of 10–120 km/h and remained nearly constant above 40 km/h. Accordingly, a rolling speed of 80 km/h was used in the subsequent simulations to evaluate the effects of pavement surface texture and *μ* on tire–pavement contact and rolling resistance.

(3) $$\begin{array}{c} R_{d}=\frac{1}{2\mathrm{\pi n}}\sum_{i=1}^{n}\Delta s_{i} \end{array}$$

where Δ*s_i_* is the displacement of the wheel center in the rolling direction during the *i*th complete rolling cycle; *n* is the number of rolling cycles, set to 5 in this study.

### 2.2. Model Validation

#### 2.2.1. Rigid Pavement Contact Validation

**Figure 5:**
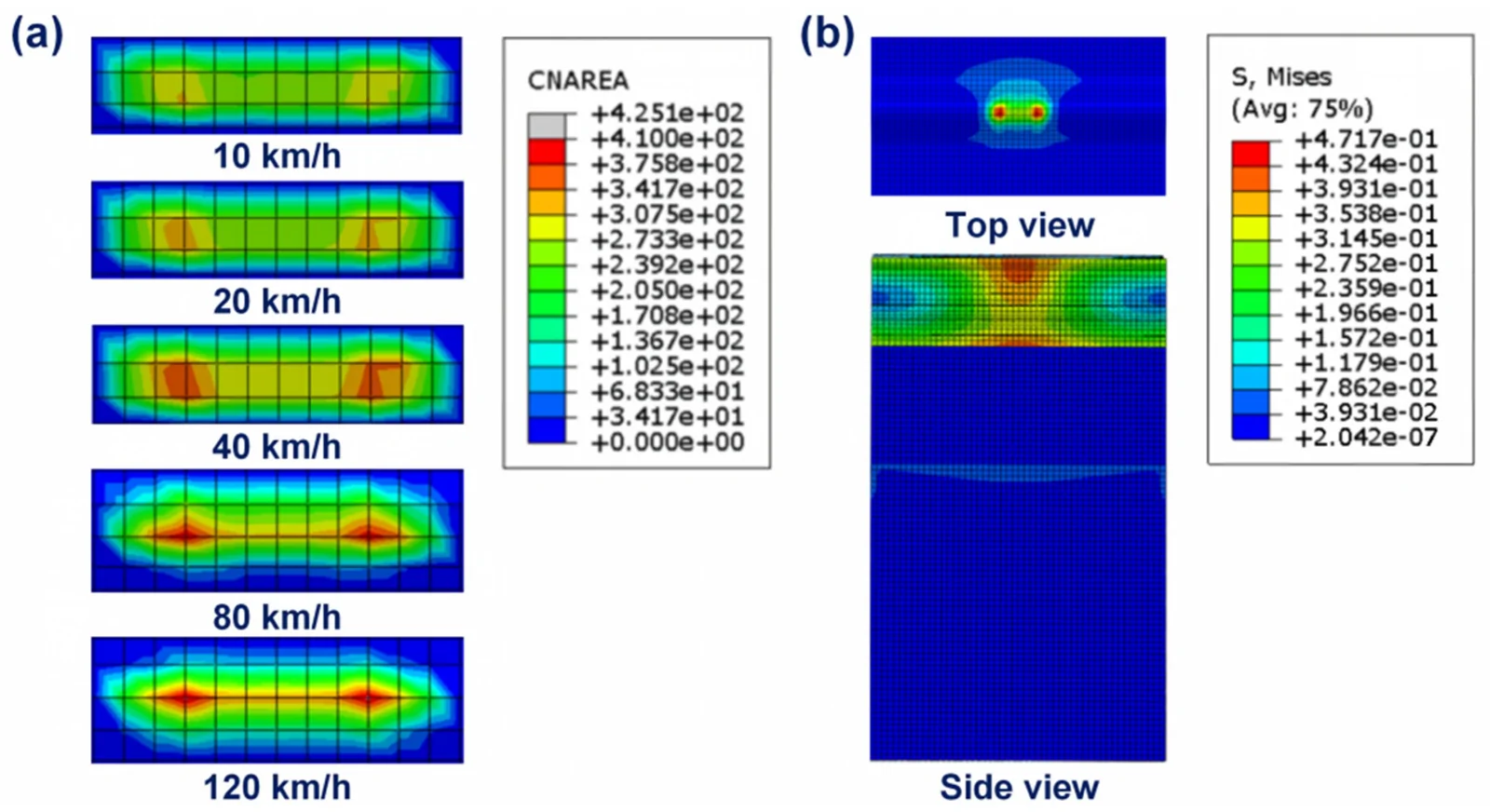
Validation of the tire–pavement interaction model: (**a**) tire–rigid pavement contact response; (**b**) tire–flexible pavement stress response.

To validate the tire–rigid pavement contact model, rolling simulations were conducted at speeds ranging from 10 to 120 km/h with the tire pressure maintained at 0.25 MPa, and the tire–pavement contact nodal area (CNAREA) distributions were extracted, as shown in Figure 5a. CNAREA represents the equivalent contact area associated with each contact node, and its spatial distribution was used to characterize the tire–pavement contact region. With increasing rolling speed, the contact area remained nearly unchanged at lower speeds but gradually decreased at higher speeds. Hernandez et al. reported that, under constant load and tire pressure, the tire contact area decreased with increasing rolling speed [25]. This trend is consistent with the present results, supporting the reliability of the developed model for characterizing tire–rigid pavement contact behavior.

#### 2.2.2. Flexible Pavement Contact Validation

Compared with the rigid pavement model, the flexible pavement model can further capture the internal mechanical response of the pavement structure under tire loading. To verify the reliability of the tire–flexible pavement model, the pavement stress distribution was extracted at a tire pressure of 0.25 MPa and a vertical load of 5000 N, as shown in Figure 5b. The results show that the high-stress region is mainly concentrated beneath the tire contact area and gradually decreases both laterally and with depth. Differences in material stiffness among the pavement layers lead to localized stress increases near some layer interfaces, reflecting the transfer and redistribution of tire loads within the layered pavement structure. Previous studies have shown that, under tire loading, pavement stresses spread from the loaded area into the underlying layers and gradually decrease with depth [26]. This trend is consistent with the present results, supporting the reliability of the model in characterizing the mechanical response of the tire–flexible pavement system.

### 2.3. Rolling Resistance Evaluation

During tire rolling, the tire undergoes continuous cyclic deformation, while tangential friction at the tire–pavement interface contributes to energy variation and dissipation. An energy-based method was used to evaluate tire rolling resistance [5,27]. Tire internal energy and interfacial frictional dissipation energy were extracted from the ABAQUS history outputs to characterize the rolling-energy response. Since this study focuses on rolling resistance arising from the tire–pavement contact system, aerodynamic drag and vehicle powertrain losses were not considered. Tire rolling resistance (*F_r_*) was calculated as the energy loss over one rolling cycle divided by the corresponding actual rolling distance, as given in Equation (4). The rolling resistance coefficient (*C_rr_*) was then calculated using Equation (5).

(4) $$\begin{array}{c} F_{r}=\frac{E_{i}+E_{f}}{2\pi R_{d}} \end{array}$$

(5) $$\begin{array}{c} C_{\mathrm{rr}}=\frac{F_{r}}{P} \end{array}$$

where *E_i_* and *E_f_* are the tire internal energy and the frictional dissipation energy at the tire–pavement interface, respectively; and *P* is the vertical tire load.

### 2.4. Reduced-Scale Rolling Resistance Test

**Figure 6:**
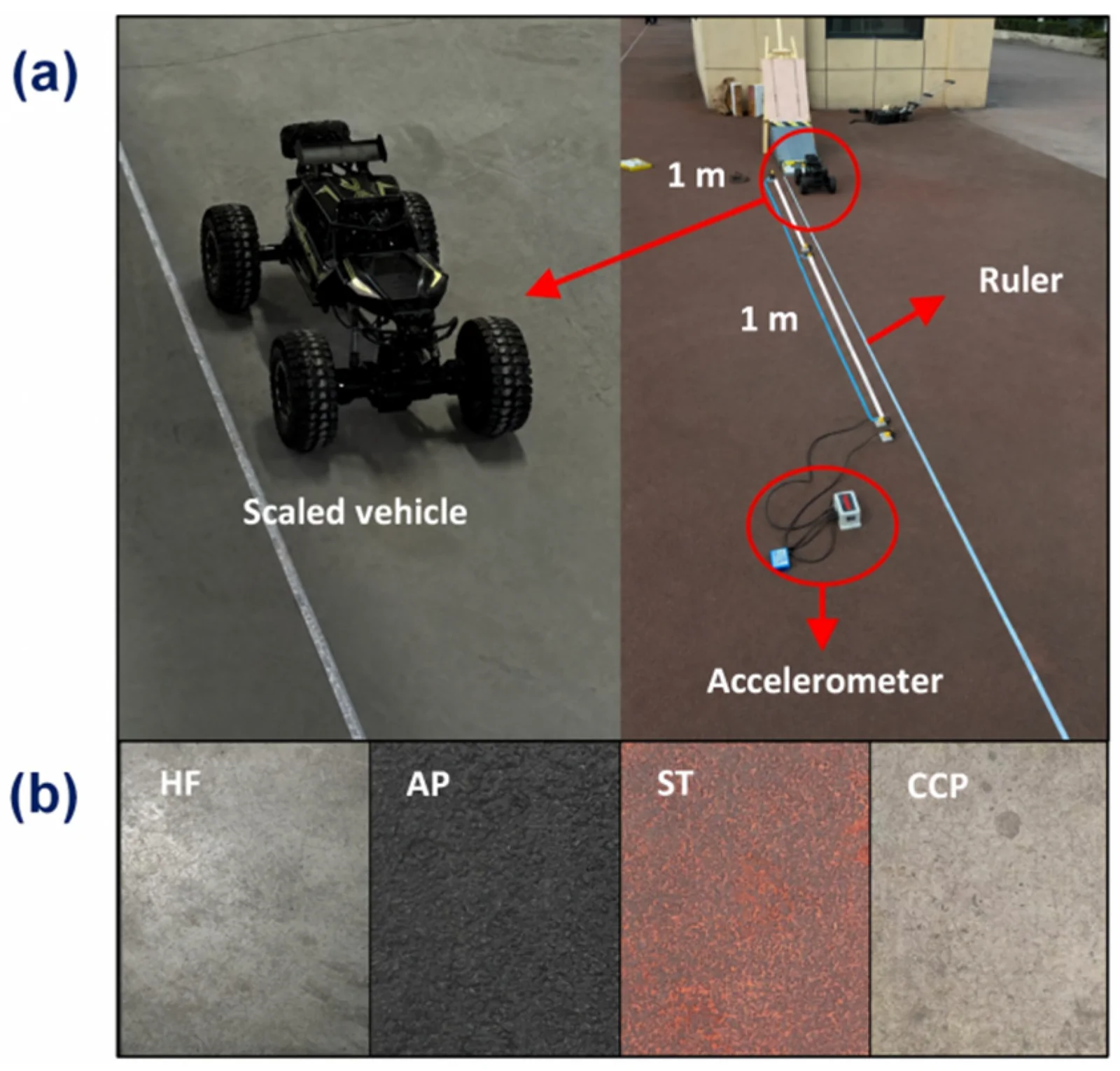
Scaled tire–pavement rolling resistance test: (**a**) test apparatus; (**b**) test surface conditions.

To investigate the effects of road surface conditions on tire rolling resistance, a scaled tire–pavement rolling resistance test apparatus was developed (Figure 6). The apparatus consisted of a test vehicle, three infrared sensors installed at 1 m intervals along the travel path, and a test platform. During each test, the vehicle was allowed to roll freely from a fixed height on the ramp, ensuring a nearly consistent initial speed upon entering the test section. Hardened flooring (HF), asphalt pavement (AP), a synthetic track (ST), and cement concrete pavement (CCP) were selected as representative surface conditions, with an additional 1 kg load placed on the vehicle to evaluate the load effect. The vehicle then coasted along the horizontal test section and gradually decelerated to a stop due to rolling resistance. Each condition was tested five times, with the initial speed (*v*_0_) and coast-down distance (*d*) recorded for each trial. Based on energy conservation, the energy balance between the initial kinetic energy (*K*_0_) of the test vehicle and the work done against rolling resistance during coasting is expressed as Equation (6).

(6) $$\begin{array}{c} K_{0}=\frac{1}{2}mv_{0}^{2}=F_{r}d=C_{\mathrm{rr}}\mathrm{mgd} \end{array}$$

Accordingly, the rolling resistance coefficient (*C_rr_*) is calculated using Equation (7).

(7) $$\begin{array}{c} C_{\mathrm{rr}}=\frac{v_{0}^{2}}{2\mathrm{gd}} \end{array}$$

where m is the vehicle mass; *F_r_* is the rolling resistance force; and *g* is the gravitational acceleration.

## 3. Results and Discussion

### 3.1. Rolling Resistance on Rigid Pavement

#### 3.1.1. Tire–Pavement Contact Response

**Figure 7:**
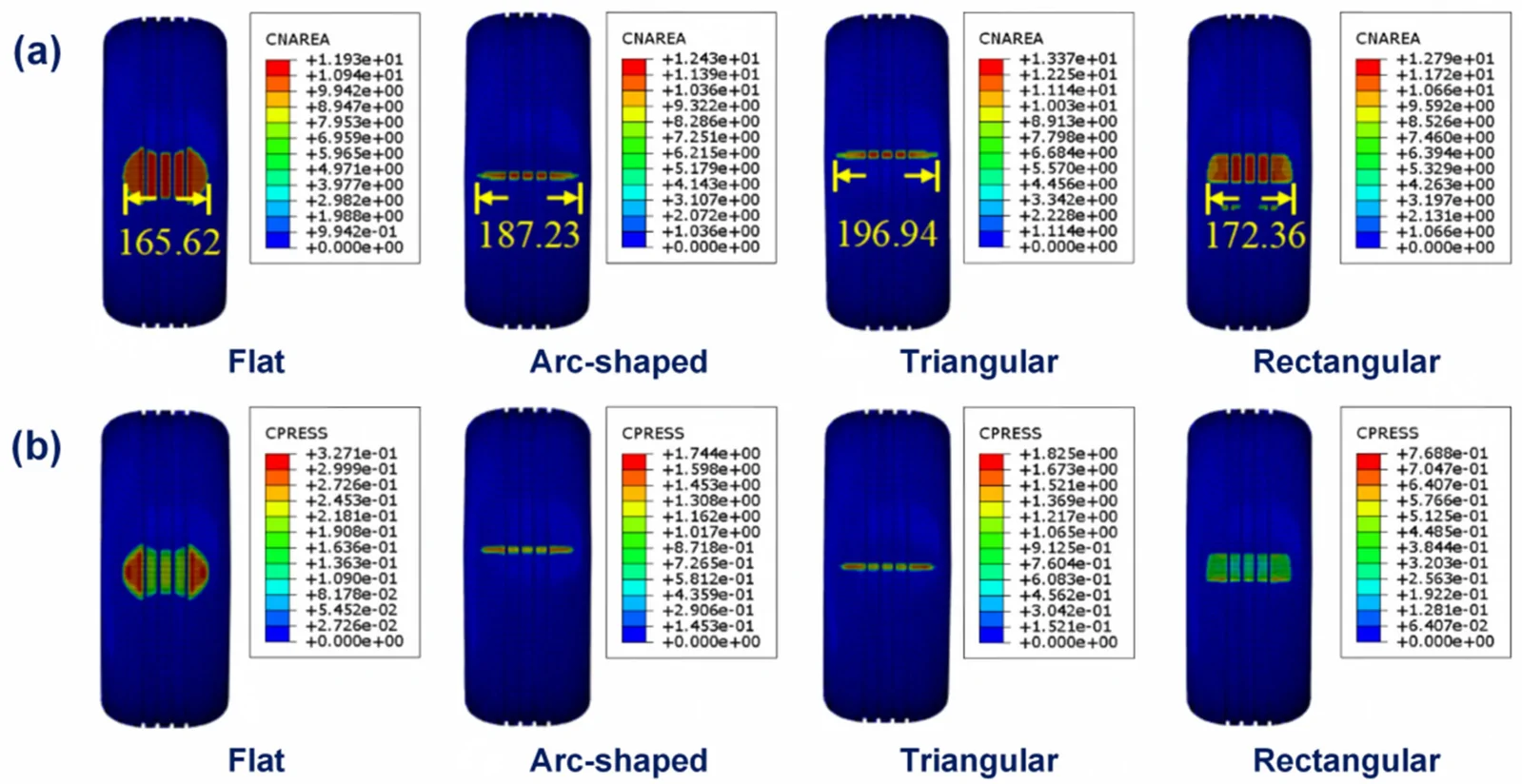
Tire–pavement contact responses under different rigid pavement surface textures (*μ* = 0.2): (**a**) contact length; (**b**) contact pressure.

To investigate the effects of rigid pavement surface texture on tire–pavement contact response and rolling resistance, simulations were performed for flat, arc-shaped, triangular, and rectangular surfaces under a vertical load of 5000 N, a tire pressure of 0.25 MPa, a friction coefficient (*μ*) of 0.2, and a rolling speed of 80 km/h. The tire–pavement contact responses during steady-state rolling are shown in Figure 7. As shown in Figure 7a, surface texture markedly influenced the tire–pavement contact region. The contact regions on the flat and rectangular surfaces were relatively broad, whereas those on the arc-shaped and triangular surfaces were more elongated. The triangular surface exhibited the greatest contact length, approximately 18.9% larger than that of the flat surface, indicating that surface texture substantially affects the extent and spatial distribution of tire–pavement contact. Figure 7b shows clear differences in contact pressure distribution among the four surface textures. Compared with the flat surface, the peak contact pressures on the arc-shaped, triangular, and rectangular surfaces increased by approximately 433.3%, 458.1%, and 135.2%, respectively. The arc-shaped and triangular surfaces produced more localized contact regions, resulting in local redistribution of the tire load and higher peak contact pressures, with the highest value observed on the triangular surface. In contrast, the rectangular surface showed a smaller increase in peak contact pressure, suggesting that the effect of surface texture on local contact pressure depends on the resulting contact extent and load distribution.

**Figure 8:**
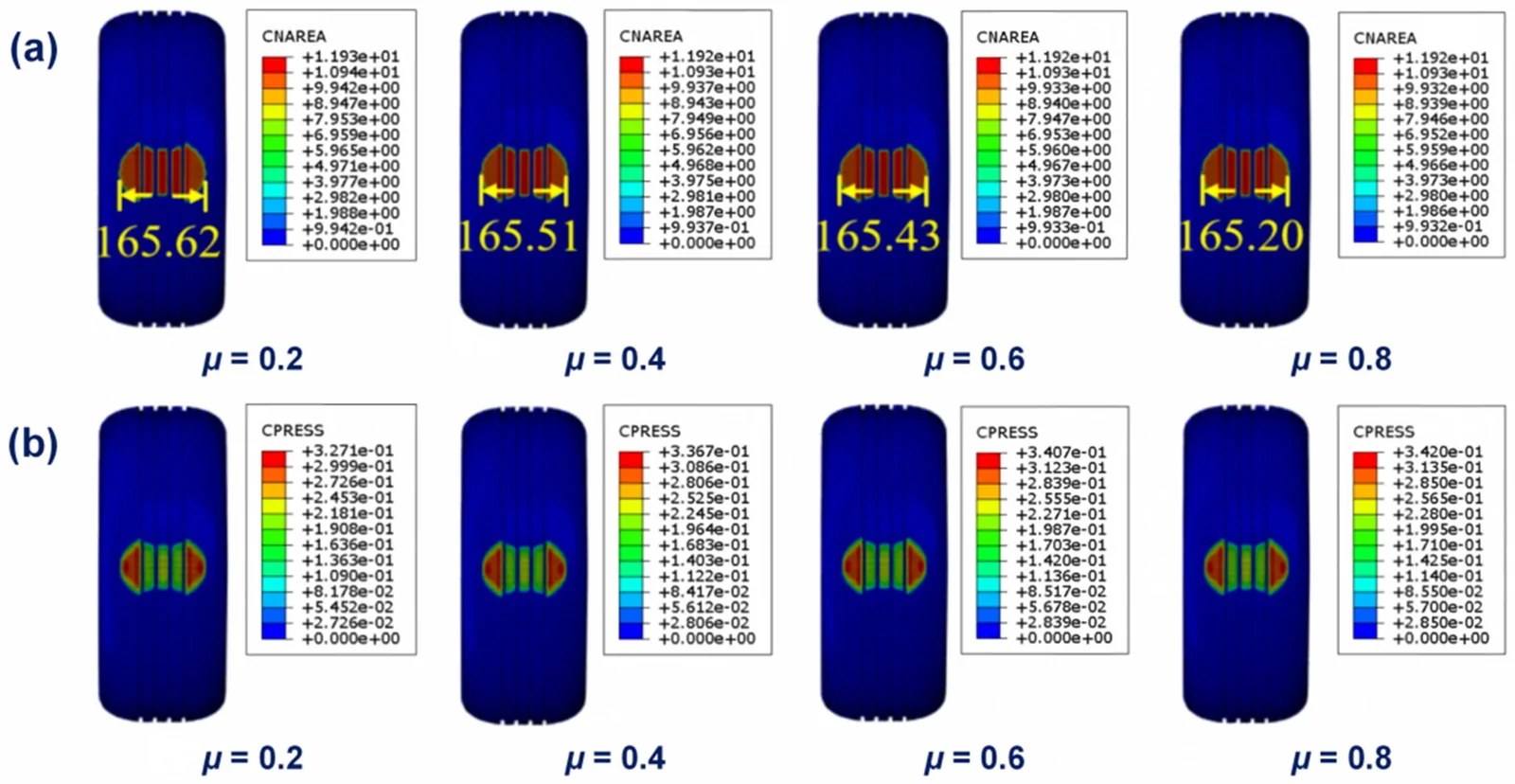
Tire–flat rigid pavement contact responses under different *μ*: (**a**) contact length; (**b**) contact pressure.

To further evaluate the effect of *μ* on tire–pavement contact response for the rigid pavement, *μ* values of 0.2, 0.4, 0.6, and 0.8 were set with all other conditions unchanged. The steady-state contact responses were then obtained. Figure 8 shows the tire–pavement contact responses on the flat rigid surface at different *μ*. As shown in Figure 8a, the contact regions remained nearly unchanged and exhibited an overall symmetric distribution with different *μ*. As *μ* increased, the contact length decreased only from 165.62 to 165.20 mm, corresponding to a reduction of approximately 0.25%, indicating a limited effect of the friction coefficient on tire–pavement contact. Figure 8b shows that the spatial distribution of contact pressure changed only slightly, while the peak contact pressure increased slightly as the friction coefficient increased. When *μ* increased from 0.2 to 0.8, the peak contact pressure increased by approximately 4.6%. This increase may be attributed to the coupling between the normal and tangential responses at the tire–pavement interface [11]. A higher *μ* strengthens the tangential interaction and alters the local normal pressure distribution through tire deformation, resulting in an increase in peak contact pressure.

**Figure 9:**
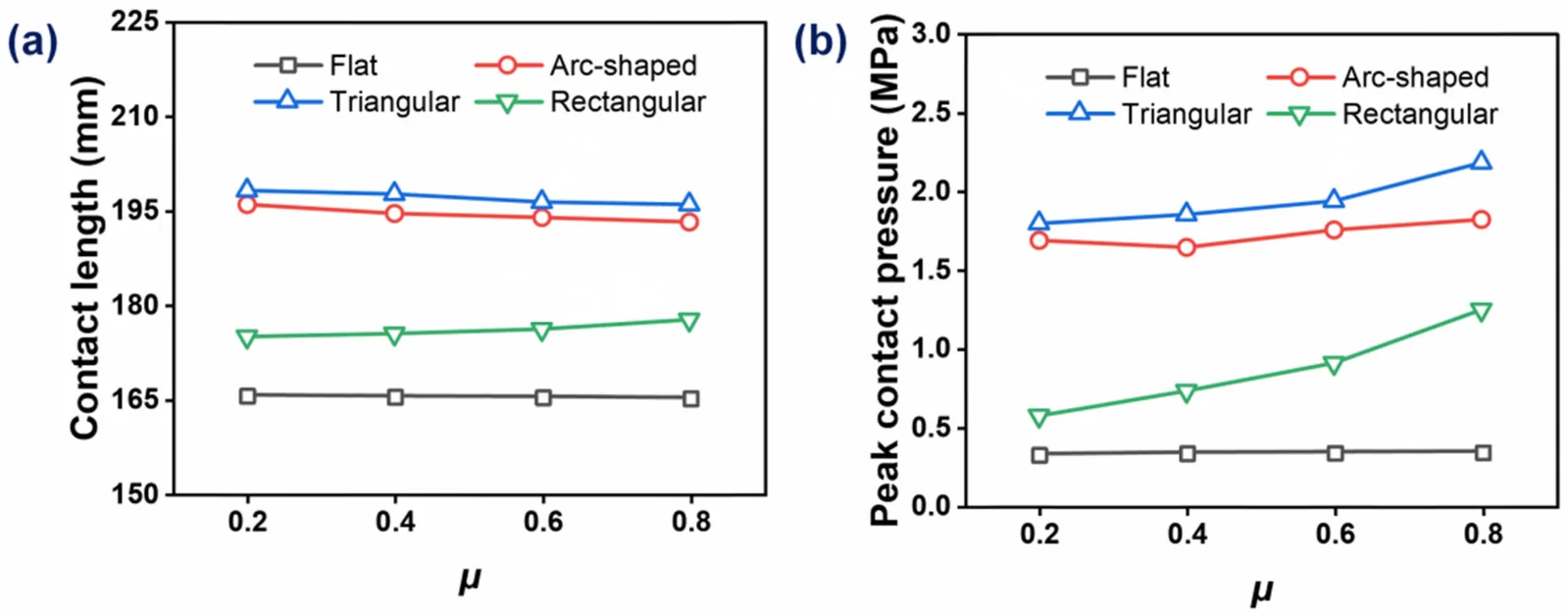
Effects of *μ* on contact responses for different rigid pavement surface textures: (**a**) contact length; (**b**) peak contact pressure.

Figure 9 shows the tire–pavement contact responses of different rigid pavement surface textures under different *μ*. As shown in Figure 9a, when *μ* increased from 0.2 to 0.8, the contact lengths of the arc-shaped and triangular surfaces decreased by approximately 1.42% and 1.14%, respectively, whereas the contact length of the rectangular surface increased by approximately 1.54%. The contact length varied only slightly among all pavement surface textures, indicating that the friction coefficient had a limited influence on the tire–pavement contact behavior. Figure 9b shows that the peak contact pressure increased as the friction coefficient increased for all surface textures. The flat surface exhibited the smallest change, whereas the peak contact pressures of the arc-shaped, triangular, and rectangular surfaces increased by approximately 7.8%, 21.6%, and 119.2%, respectively, as the friction coefficient increased from 0.2 to 0.8. The increase in peak contact pressure with the friction coefficient indicates that frictional interaction can modify the tire–pavement contact behavior. Such changes in contact pressure may further affect local tire deformation and energy dissipation during rolling, which can contribute to variations in rolling resistance.

#### 3.1.2. Rolling Resistance Under Different Surface Textures and μ

**Figure 10:**
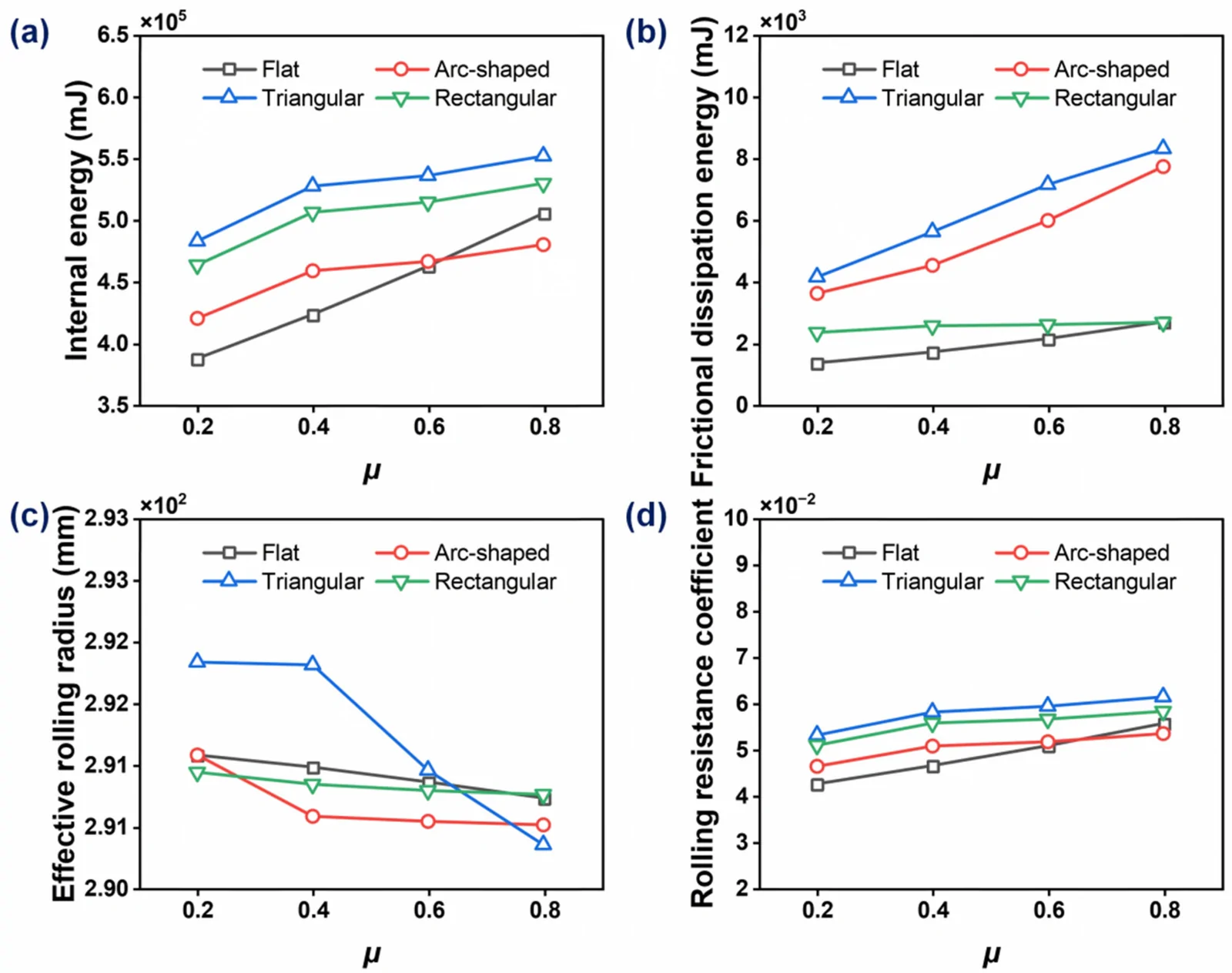
Tire rolling behavior under different rigid pavement surface textures: (**a**) internal energy; (**b**) friction dissipated energy; (**c**) effective rolling radius; (**d**) rolling resistance coefficient.

Figure 10 presents the energy dissipation characteristics and rolling resistance of the tire under different *μ* and pavement surface textures during rolling. Figure 10a,b show that the tire internal energy (*E_i_*) and interfacial frictional dissipation energy (*E_f_*) increased with increasing *μ* for all pavement surface textures. As *μ* increased from 0.2 to 0.8, *E_i_* and *E_f_* increased by about 14.3–30.5% and 14.3–114.7%, respectively, indicating greater tire deformation-related and friction-induced energy dissipation. Figure 10c shows that the effective rolling radius (*R_d_*) remained relatively stable as *μ* increased for different pavement surface textures, with a maximum variation of about 0.6%. This indicates that *μ* and pavement surface textures have limited effects on *R_d_*, whereas variations in rolling resistance are primarily associated with energy dissipation due to tire deformation and tire–pavement interfacial friction.

The rolling resistance coefficient (*C_rr_*) was calculated using Equation (4). As shown in Figure 10d, *C_rr_* increased as *μ* increased for different pavement surface textures. As *μ* increased from 0.2 to 0.8, *C_rr_* of the flat, arc-shaped, triangular, and rectangular surface textures increased by approximately 30.8%, 15.3%, 15.6%, and 14.5%, respectively. The flat surface showed the largest increase in *C_rr_*, whereas the triangular surface showed the highest *C_rr_*, approximately 10.4–24.9% higher than that of the flat surface. This is mainly attributed to its pronounced geometric features, which induce greater local tire deformation and frictional dissipation during tire–pavement contact, resulting in higher energy loss and rolling resistance. In contrast, the flat surface provides a more uniform contact condition, with relatively lower tire deformation and interfacial frictional dissipation, resulting in lower rolling resistance.

### 3.2. Rolling Resistance on Flexible Pavement

#### 3.2.1. Tire–Pavement Contact Response

**Figure 11:**
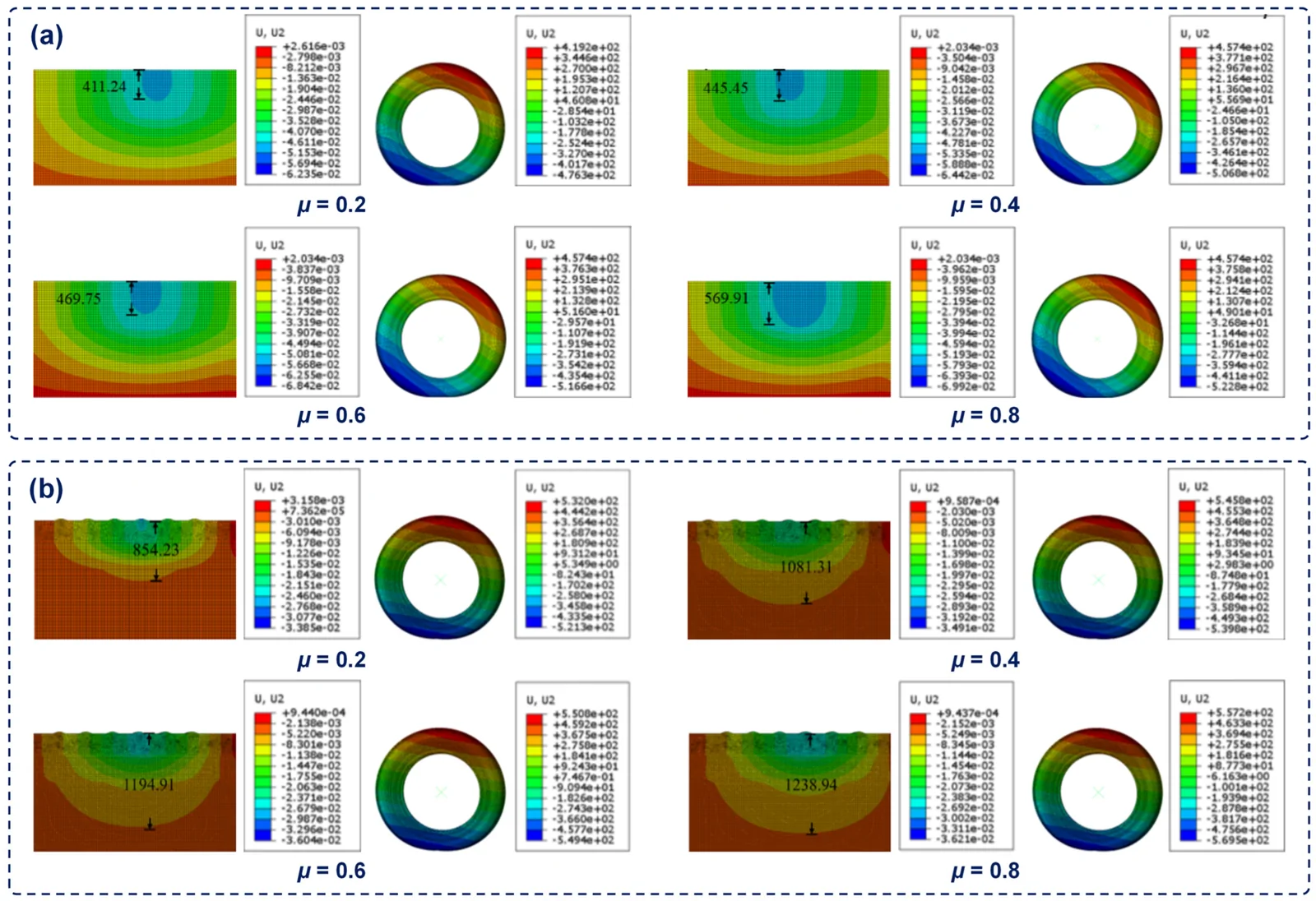
Vertical displacement distributions of flexible pavement and tire under different *μ*: (**a**) flat pavement; (**b**) arc-shaped pavement.

Compared with the rigid pavement model, the flexible pavement model accounts for structural deformation under tire loading, which further influences tire–pavement contact behavior. Figure 11 presents the vertical displacement distributions of the tire and flexible pavement surfaces with flat and arc-shaped textures under different *μ*. The regions with high vertical displacement in the tire and pavement are mainly concentrated within the tire–pavement contact area and gradually expand as *μ* increases, indicating that higher friction leads to a more pronounced deformation response. Relative to the flat surface, the arc-shaped texture results in a larger high-displacement region, indicating that pavement surface textures influence deformation transfer within flexible pavement.

**Figure 12:**
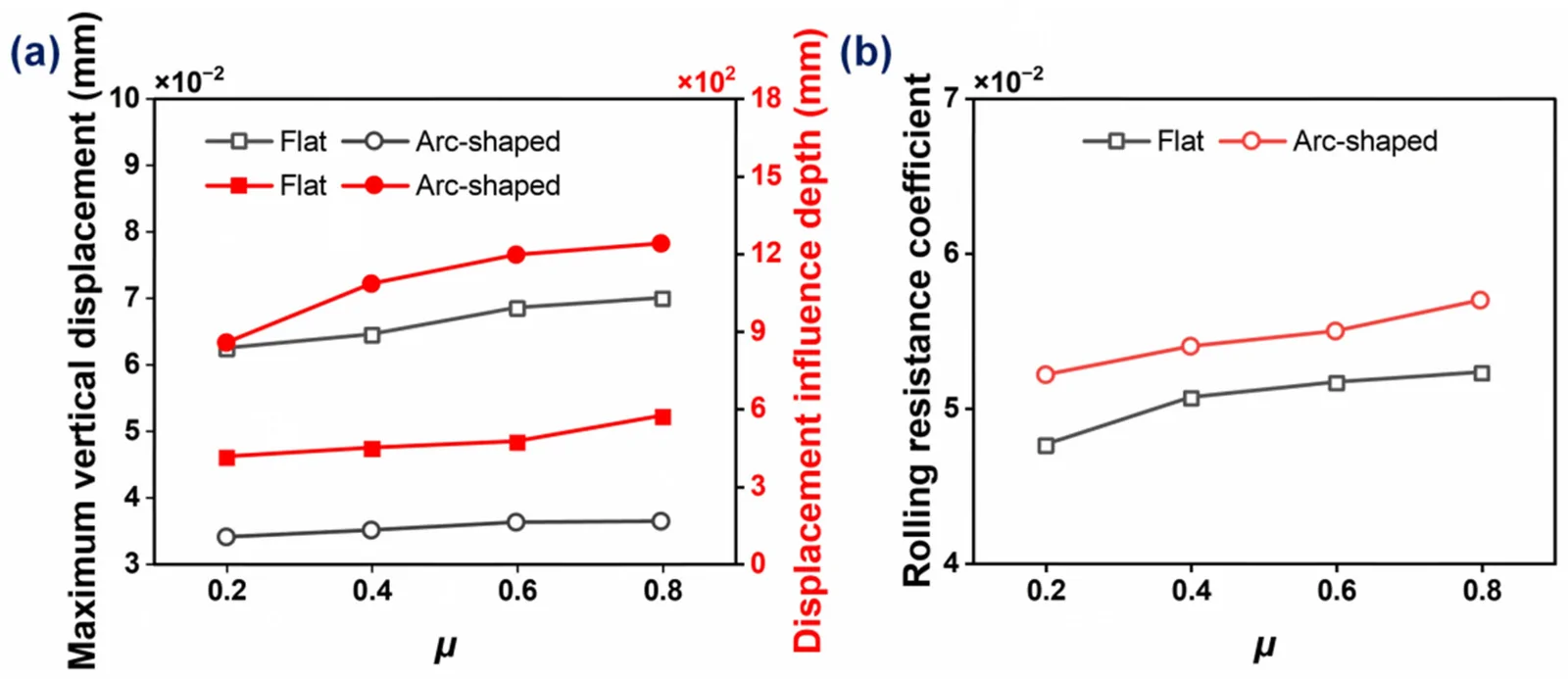
Vertical deformation response under different *μ*: (**a**) flexible pavement; (**b**) tire.

To further quantify the vertical deformation response, Figure 12 presents the maximum vertical displacement of the flexible pavement and tire under different pavement surface textures. As *μ* increased from 0.2 to 0.8, the maximum vertical displacement and displacement influence depth increased by approximately 12.1% and 38.6% for the flat surface, respectively, and by approximately 7.0% and 45.0% for the arc-shaped surface, respectively. The larger increase in displacement influence depth than in maximum vertical displacement indicates that increasing friction coefficient has a stronger effect on the expansion of deformation range within flexible pavement. Meanwhile, the maximum vertical displacement of the tire also increased as the friction coefficient increased, by approximately 9.8% and 7.9% for the flat and arc-shaped surfaces, respectively. The arc-shaped surface consistently exhibited a higher vertical deformation response. This can be attributed to the curved surface geometry, which modifies local tire–pavement contact and load distribution, resulting in greater deformation. Previous studies have also shown that pavement surface geometry can significantly influence local contact conditions and tread deformation [28]. These results suggest that an increase in friction coefficient strengthens tire–pavement interaction, leading to increased local tire deformation and an expanded deformation range within flexible pavement. In addition, pavement surface textures influence deformation transfer, with the arc-shaped texture producing a more pronounced coupled deformation response.

#### 3.2.2. Rolling Resistance Under Different Surface Textures and *μ*

**Figure 13:**
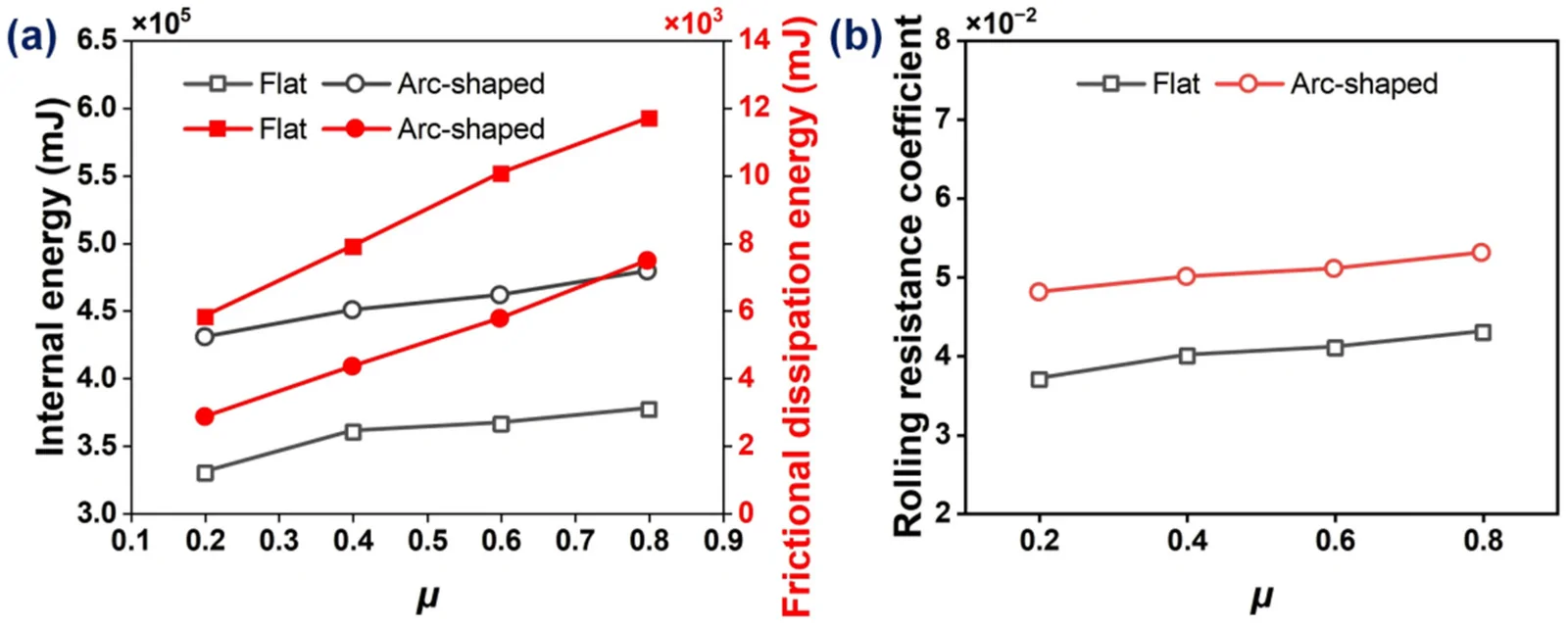
Tire rolling responses under different *μ*: (**a**) internal energy and friction dissipated energy; (**b**) rolling resistance coefficient.

To investigate the effects of *μ* and pavement surface textures on rolling resistance under flexible pavement conditions, Figure 13 presents the energy dissipation characteristics during tire rolling and the corresponding rolling resistance coefficients (*C_rr_*) obtained using Equation (4). As shown in Figure 13a, the tire internal energy (*E_i_*) and interfacial frictional dissipation energy (*E_f_*) increased as *μ* increased from 0.2 to 0.8 for both flat and arc-shaped surfaces. For the flat surface, *E_i_* and *E_f_* increased by approximately 14.3% and 100.8%, respectively. For the arc-shaped pavement surface, these increases were approximately 11.4% and 163.2%, respectively. This indicates that an increase in *μ* enhances tire–pavement interaction, thereby increasing energy dissipation associated with tire deformation and interfacial friction during rolling. This trend is consistent with that observed for rigid pavement.

Figure 13b shows that *C_rr_* increased as *μ* increased for different pavement surface textures. As *μ* increases from 0.2 to 0.8, *C_rr_* of the flat and arc-shaped pavements increases by approximately 16.2% and 10.4%, respectively. Compared with the flat surface, the arc-shaped surface exhibited a higher *C_rr_*, indicating that surface texture affects tire–pavement interaction and increases energy dissipation. This difference can be mainly attributed to the more pronounced deformation response induced by the arc-shaped texture, as shown in Figure 12b. Previous studies have shown that pavement surface texture can induce local tread deformation, which contributes to rolling energy loss and is closely related to surface geometric characteristics [28]. Therefore, the greater deformation observed for the arc-shaped texture results in increased energy dissipation during rolling and, consequently, a higher rolling resistance coefficient.

### 3.3. Rolling Resistance Test Results

**Figure 14:**
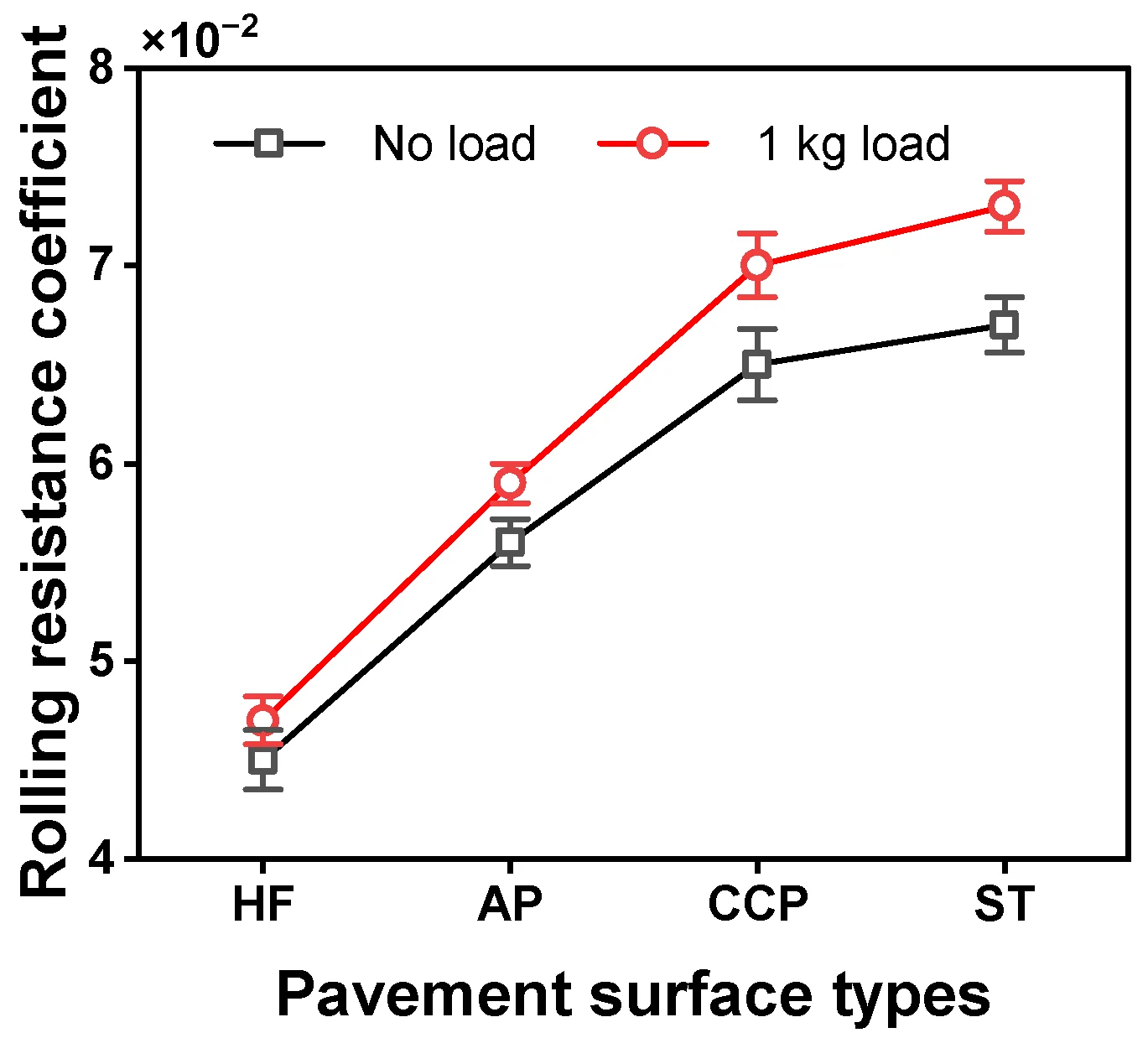
Rolling resistance coefficients under different pavement types.

To validate the finite element simulation results and investigate the effect of pavement surface friction characteristics on tire rolling resistance, scaled rolling resistance tests were conducted on different pavement surfaces, with five repetitions performed for each surface condition. To evaluate the surface friction characteristics of different pavement surfaces, the British Pendulum Number (BPN) values of each pavement surface were measured using a British pendulum tester. The BPN values of different pavement surfaces were ranked as follows: synthetic track (69) > cement concrete pavement (63) > asphalt pavement (52) > hardened flooring (48). BPN was used to indicate the relative friction level among the tested pavement surfaces, rather than to quantitatively represent *μ*. *C_rr_* values for different pavement surfaces calculated using Equation (5) are shown in Figure 14. Error bars represent the standard deviations of five repeated measurements. *C_rr_* showed the same ranking as BPN, with the highest value observed for the synthetic track (ST), followed by cement concrete pavement (CCP), asphalt pavement (AP), and hardened flooring (HF). Without additional load, *C_rr_* increased from approximately 0.045 for hardened flooring to approximately 0.067 for synthetic track. After applying an additional load of 1 kg, *C_rr_* further increased for all pavement surfaces, reaching approximately 0.073 for the synthetic track, while the hardened flooring maintained the lowest value. These results suggest that pavement surface friction characteristics play an important role in determining rolling resistance. A higher pavement surface friction level enhances tangential interaction at the tire–pavement interface, leading to greater energy dissipation due to tire deformation and interfacial friction during rolling and consequently a higher *C_rr_*. The observed trend is qualitatively consistent with the finite element results, providing further support for the conclusion that higher tire–pavement interfacial friction increases rolling resistance.

## 4. Conclusions

To investigate the effects of pavement surface textures and friction coefficients on tire–pavement contact behavior and rolling resistance, a three-dimensional tire–pavement rolling finite element model was developed. The contact responses, energy dissipation, and rolling resistance responses under different surface textures and friction coefficients were investigated, and scaled rolling resistance tests were conducted for qualitative comparison with the simulation results. The main conclusions are as follows:

(1) Pavement surface textures and tire–pavement interfacial friction influence rolling resistance through their effects on contact response and energy dissipation. Under rigid pavement conditions, the triangular texture induces stronger local contact responses than the flat texture, increasing the contact length and peak contact pressure by approximately 18.9% and 458.1%, respectively. Increasing *μ* from 0.2 to 0.8 promotes greater energy dissipation caused by tire deformation and interfacial friction, thereby increasing rolling resistance.
(2) Compared with rigid pavement, structural deformation of flexible pavement contributes to energy dissipation during tire rolling. As *μ* increases, tire and pavement deformation responses become more pronounced, the deformation range expands, and energy dissipation caused by tire deformation and interfacial friction increases, thereby increasing *C_rr_*. The arc-shaped texture exhibits the most pronounced deformation response and the highest *C_rr_*.
(3) The scaled rolling resistance tests showed qualitative consistency with the finite element results regarding the effect of pavement surface friction characteristics on rolling resistance. *C_rr_* of different pavement surfaces increased with increasing surface friction level, and additional loading further increased *C_rr_*. These results further supported the observed trend that tire–pavement interfacial friction influences rolling resistance.

## Figures and Tables

**Table 1:** Parameters of tire rubber materials.

Type	Yeoh Model Parameters			Density (ton/mm^3^)
	*C*_10_	*C*_20_	*C*_30_	
Tread rubber	0.751	−0.0376	0.07930	1.14 × 10^−9^
Belt rubber	4.621	−0.071	10.026	1.18 × 10^−9^
Inner rubber	0.546	−0.082	0.0138	1.15 × 10^−9^
Bead rubber	1.664	−0.088	0.101	1.17 × 10^−9^
Sidewall rubber	0.392	−0.0943	−0.0296	1.09 × 10^−9^
Triangle rubber	1.953	−0.0415	0.0074	1.11 × 10^−9^

**Table 2:** Parameters of tire reinforcement materials.

Type	Cross-Sectional Area (mm^2^)	Orientation Angle (°)	Spacing (mm)	Young’s Modulus (MPa)	Poisson’s Ratio	Density (ton/mm^3^)
Upper belt ply	1.2	70	1	12,000	0.3	4.8 × 10^−9^
Lower belt ply	1.2	−70	1	12,000	0.3	4.8 × 10^−9^
Steel wire	/	/	/	39,000	0.3	4.8 × 10^−9^

**Table 3:** Parameters of the flexible pavement model.

Pavement Structure	Materials	Thickness (mm)	Modulus (MPa)	Poisson’s Ratio
Surface layer	Asphalt mixture	300	4500	0.3
Base layer	Graded crushed stone	400	500	0.35
Subgrade	Backfill soil	1000	50	0.4

## Data Availability

The original contributions presented in this study are included in the article. Further inquiries can be directed to the corresponding author.

## References

[1] Shah K., Pan S.-Y., Lee I., Kim H., You Z., Zheng J.-M., Chiang P.-C. (2021). Green transportation for sustainability: Review of current barriers, strategies, and innovative technologies. J. Clean. Prod..

[2] Steyn W., Lombard D., Mashabela G., Rudolph J., Francois W. Vehicle Rolling Resistance as Affected by Tire and Road Conditions. Proceedings of the 4th Geo-China International Conference.

[3] Wang Q., Li A., Yan J. (2025). Study on the relationship between the steady-state rolling resistance of radial tires and energy consumption of electric vehicles. Sci. Rep..

[4] Ding Y., Wang H., Qian J., Zhou H. (2021). Evaluation of Tire Rolling Resistance from Tire-Deformable Pavement Interaction Modeling. J. Transp. Eng. Part B Pavements.

[5] Cho J., Lee H., Jeong W., Jeong K., Kim K.-W. (2013). Finite element estimation of hysteretic loss and rolling resistance of 3-D patterned tire. Int. J. Mech. Mater. Des..

[6] Liu Q., Pei J., Wang Z., Hu D., Huang G., Meng Y., Lyu L., Zheng F. (2024). Analysis of tire-pavement interaction modeling and rolling energy consumption based on finite element simulation. Constr. Build. Mater..

[7] Li F., Liu F., Liu J., Gao Y., Lu Y., Chen J., Yang H., Zhang L. (2018). Thermo-mechanical coupling analysis of transient temperature and rolling resistance for solid rubber tire: Numerical simulation and experimental verification. Compos. Sci. Technol..

[8] Aldhufairi H., Olatunbosun O. (2017). Developments in tyre design for lower rolling resistance: A state of the art review. Proc. Inst. Mech. Eng. Part D J. Automob. Eng..

[9] Zhang Q., Shangguan L., Li T., Ma X., Yin Y., Dong Z. (2023). Tire–Pavement Interaction Simulation Based on Finite Element Model and Response Surface Methodology. Computation.

[10] Rafei M., Ghoreishy M.H.R., Naderi G. (2018). Computer simulation of tire rolling resistance using finite element method: Effect of linear and nonlinear viscoelastic models. Proc. Inst. Mech. Eng. Part D J. Automob. Eng..

[11] Hernandez J., Al-Qadi I. (2016). Tire–pavement interaction modelling: Hyperelastic tire and elastic pavement. Road Mater. Pavement Des..

[12] Suvanjumrat C., Rugsaj R. (2017). Finite Element Modeling with Embed Rebar Elements and Steady State Rolling Analysis for Rolling Resistance Test of Pneumatic Tire. MATEC Web Conf..

[13] Curtosi G., Zitelli P., Kuster J. (2019). Viscoelastic Material Calibration Procedure for Rolling Resistance Calculation. Tire Sci. Technol..

[14] Swieczko-Zurek B., Ronowski G., Ejsmont J. (2017). Tyre rolling resistance and its influence on fuel consumption. Combust. Engines.

[15] Andersen L., Larsen J., Fraser E., Schmidt B., Dyre J. (2015). Rolling Resistance Measurement and Model Development. J. Transp. Eng..

[16] Yam L.H., Shang J., Guan D.H., Zhang A.Q. (2002). Study on tyre rolling resistance using experimental modal analysis. Int. J. Veh. Des..

[17] Ghosh P., Tomer A., Mukhopadhyay S., Mukhopadhyay R. (2021). Influence of Tyre Rolling Resistance and Operational Parameters on Vehicle Fuel Efficiency.

[18] Kouchaki S., Roshani H., Prozzi J., Zuniga-Garcia N., Hernandez J. (2018). Field Investigation of Relationship between Pavement Surface Texture and Friction. Transp. Res. Rec. J. Transp. Res. Board.

[19] Edmondson V., Woodward J., Lim M., Kane M., Martin J., Shyha I. (2019). Improved non-contact 3D field and processing techniques to achieve macrotexture characterisation of pavements. Constr. Build. Mater..

[20] Kogbara R., Masad E., Kassem E., Scarpas T., Anupam K. (2016). A state-of-the-art review of parameters influencing measurement and modeling of skid resistance of asphalt pavements. Constr. Build. Mater..

[21] Peng Y., Li J., Zhan Y., Wang K., Yang G. (2019). Finite Element Method-Based Skid Resistance Simulation Using In-Situ 3D Pavement Surface Texture and Friction Data. Materials.

[22] Zheng B., Chen J., Zhao R., Tang J., Tian R., Zhu S., Huang X. (2020). Analysis of contact behaviour on patterned tire-asphalt pavement with 3-D FEM contact model. Int. J. Pavement Eng..

[23] Meng Y., Zheng F., Pei J., Dai K., Liu Q., Hu D., Kingan M. (2025). Study on tire-pavement contact characteristics and tire vibration noise based on finite element numerical simulation. Int. J. Pavement Eng..

[24] Dan H.-C., Lu B., Li M. (2024). Evaluation of asphalt pavement texture using multiview stereo reconstruction based on deep learning. Constr. Build. Mater..

[25] Hernandez J., Al-Qadi I. (2015). Hyperelastic Modeling of Wide-Base Tire and Prediction of Its Contact Stresses. J. Eng. Mech..

[26] Al-Qadi I.L., Loulizi A., Janajreh I., Freeman T.E. (2002). Pavement Response to Dual Tires and New Wide-Base Tires at Same Tire Pressure. Transp. Res. Rec. J. Transp. Res. Board.

[27] Sutisna N. (2020). Rolling Resistance Estimation for PCR Tyre Design Using the Finite Element Method. Finite Element Methods and Their Applications.

[28] Hoever C., Kropp W. (2015). A model for investigating the influence of road surface texture and tyre tread pattern on rolling resistance. J. Sound Vib..

